# Assessment of performance of survival prediction models for cancer prognosis

**DOI:** 10.1186/1471-2288-12-102

**Published:** 2012-07-23

**Authors:** Hung-Chia Chen, Ralph L Kodell, Kuang Fu Cheng, James J Chen

**Affiliations:** 1 Division of Bioinformatics and Biostatistics, National Center for Toxicological Research, U.S. Food and Drug Administration, 3900 NCTR Road, HFT-20, Jefferson, AR, 72079, USA; 2 Department of Biostatistics, University of Arkansas for Medical Sciences, 4301 West Markham Street, #781, Little Rock, AR, 72205, USA; 3 Biostatistics Center, School of Public Health, China Medical University, Taichung, Taiwan

## Abstract

**Background:**

Cancer survival studies are commonly analyzed using survival-time prediction models for cancer prognosis. A number of different performance metrics are used to ascertain the concordance between the predicted risk score of each patient and the actual survival time, but these metrics can sometimes conflict. Alternatively, patients are sometimes divided into two classes according to a survival-time threshold, and binary classifiers are applied to predict each patient’s class. Although this approach has several drawbacks, it does provide natural performance metrics such as positive and negative predictive values to enable unambiguous assessments.

**Methods:**

We compare the survival-time prediction and survival-time threshold approaches to analyzing cancer survival studies. We review and compare common performance metrics for the two approaches. We present new randomization tests and cross-validation methods to enable unambiguous statistical inferences for several performance metrics used with the survival-time prediction approach. We consider five survival prediction models consisting of one clinical model, two gene expression models, and two models from combinations of clinical and gene expression models.

**Results:**

A public breast cancer dataset was used to compare several performance metrics using five prediction models. 1) For some prediction models, the hazard ratio from fitting a Cox proportional hazards model was significant, but the two-group comparison was insignificant, and *vice versa*. 2) The randomization test and cross-validation were generally consistent with the p-values obtained from the standard performance metrics. 3) Binary classifiers highly depended on how the risk groups were defined; a slight change of the survival threshold for assignment of classes led to very different prediction results.

**Conclusions:**

1) Different performance metrics for evaluation of a survival prediction model may give different conclusions in its discriminatory ability. 2) Evaluation using a high-risk versus low-risk group comparison depends on the selected risk-score threshold; a plot of p-values from all possible thresholds can show the sensitivity of the threshold selection. 3) A randomization test of the significance of Somers’ rank correlation can be used for further evaluation of performance of a prediction model. 4) The cross-validated power of survival prediction models decreases as the training and test sets become less balanced.

## Background

The Cox proportional hazards model [1] is the most common survival prediction model for cancer prognosis. Often, demographic and clinical covariates are combined in a Cox model with staging information from the American Joint Committee on Cancer (AJCC) staging system to predict a patient’s survival to improve treatment recommendations [2-7]. Because microarray studies have shown an association between patient survival and gene expression profiles [8-10], some recent papers have investigated the use of microarray gene expression data alone or in combination with clinical covariates [11-14] as an improvement to estimate patient survival risk. Dimensionality reduction techniques are often performed prior to applying the Cox model to improve prediction performance. A practical approach is to apply a selection technique to select a smaller set of relevant genes from the entire gene set as initial step; a dimensionality reduction technique is then applied to the selected gene set [15].

Evaluation of the ability of a survival model to predict future data is the most important consideration in the development of prediction model. Common metrics to assess the performance of survival prediction models include hazard ratios between high- and low-risk groups defined by dichotomized risk scores, and tests for significant differences in the two groups’ Kaplan-Meier survival curves. Metrics that measure the strength of the relationship between risk scores and survival, including the simple hazard ratio, the coefficient of determination R^2^[16], the concordance index and Somers’ rank correlation D_xy_[17,18], are also used. Additional metrics include receiver operating characteristic (ROC) curves [19] and area under the ROC curve (AUC) [20-22] defined over a range of risk-score cutoffs given a fixed survival threshold. Hielscher et al. [23] recently compared several common R^2^-type measures for evaluation of survival models. In addition, the Brier scores and Schemper/Henderson measure which were also developed to assess the survival prediction models were investigated by Schumacher et al. [24] and Dunkler et al. [25], respectively. To our knowledge no comprehensive evaluation and comparison among different performance metrics for different survival modeling approaches has been reported.

In contrast to the commonly used survival-time prediction model approach, much research in gene expression profiling of cancer data has focused on binary class prediction, where patients’ survival times have been dichotomized to form two classes [11,23,26-37]. With this approach, a prediction model is built and used to distinguish between the “low-risk” and ‘high-risk” classes. The performance of a binary classifier is generally evaluated in terms of the overall predictive accuracy, along with positive and negative predictive values, etc. [28]. Dupuy and Simon [27] discussed several drawbacks of using this approach with survival data. Mainly, it does not take the information on survival and censored times into consideration. Furthermore, binary classification highly depends on the survival threshold used to define the two classes. A slight change of the threshold can lead to very different prediction accuracy and interpretation. Binder et al. [30] applied three different survival thresholds to evaluate a binary classifier based on gene expression, and showed how the choice of threshold affected the predictions. They concluded that using the binary modeling approach can result in loss of efficiency and potential bias in high dimensional settings.

In this study, we evaluate and compare commonly used metrics to assess performance of five prediction models. These five models are based on established approaches to modeling clinical variables and microarray gene expression data. We propose using randomization tests to compute the p-values of certain performance metrics, including D_xy_, R^2^, and AUC, and cross-validation to evaluate the power of the prediction models. We also present an analysis to illustrate differences between the survival-time threshold (binary classification) and survival-time prediction (survival risk score) models in the analysis of survival data.

## Methods

### Models for survival outcomes developed from training dataset

Five survival prediction models to estimate patient’s survival risks were considered. These five models included one clinical model, two gene expression models, and two models based on the combinations of the clinical and two gene expression models.

The clinical model (Model A) was the Cox proportional hazards model, derived by fitting the Cox model to all clinical variables, TNM stage, gender, age, and others, and selecting the clinical variables most relevant to the training dataset. The gene expression data were first analyzed using the univariate Cox model to select a set of “significant” genes, based on a pre-determined statistical criterion which is p < 0.001 in this paper. For the set of selected genes, two gene expression models were developed using the Cox model: Model B used the first five principal components of the set of significant genes as signature variables, and Model C used the top 10 ranked genes as signature predictors. Each of the gene expression models was combined with the clinical model (Model A) additively to develop two clinical and gene expression models (D = A + C and E = A + D). A summary of the five models is given in Additional file 1: Table S1.

### Assessment of predicted risk scores for the patients in test dataset

The regression coefficients of the fitted Cox models (A-E) developed from the training data were used to compute the predictive risk scores for each patient in the test dataset. The predictive risk scores were then used to compute performance metrics to evaluate the performance of the prediction models built from the training data. We considered the following commonly used performance metrics. A more detailed description of these metrics is given in the Additional file 1.

### Simple hazard ratio and R^2^ (Cox Model I)

A Cox model was fit using the predictive risk scores as an independent variable with survival time as the outcome variable. The exponent of the regression coefficient was the simple hazard ratio. The performance metrics included: estimated hazard ratio, 95% confidence limits on hazard ratio, p-value for significance of hazard ratio, and R^2^[23,38].

### Two-group hazard ratio and brier score (Cox Model II)

The test data were first segregated into high-risk and low-risk groups by the median of training risk scores. A Cox model was fit using the risk group as an independent variable with survival time as the outcome variable. The exponent of the regression coefficient was the two-group hazard ratio. The performance metrics included the estimated hazard ratio, 95% confidence limits on the hazard ratio, and p-value for significance of hazard ratio.

The Brier score [31], which measures average discrepancies between true disease status and estimated predictive values, was also calculated to assess the predictive risk scores in risk group stratification. The Brier score can be calculated for a specific time point or for an overall error measure across all time points. A larger Brier score means a higher inaccuracy of a prognostic classification scheme. However, baseline estimation is required for computing predicted risk-free probability to estimate Brier score, and different methods used could result in different Brier scores. Therefore we applied the method developed by Graf et al. [31] to compute integrated Brier score (IBS) in the two-group stratification without baseline estimation, where the test data are stratified into two groups according to the training model and the risk-free probability for each sample is estimated from the Kaplan-Meier estimate for the corresponding group.

### Log-rank test

The log-rank test was used to compare the survival curves between the patients in the high risk and low risk groups defined by the predicted risk scores. The performance metric was the p-value of the test.

### Somers’ rank correlation D_xy_

The concordance index between predicted risk score and observed survival time in the test dataset was computed by a rank correlation adjusted for censored time [17,18]. This index was re-expressed equivalently as a correlation measure, known as the Somers’ D_xy_ rank correlation. The performance metrics included the calculated D_xy_ and the p-value of a randomization test of its significance.

### Time dependent Receiver Operating Characteristic (ROC) Curve and the Area under the ROC Curve (AUC)

For a given survival threshold, t, ROC(t) was plotted as sensitivity(t) versus 1-specificity(t) for all values of the risk score cutoff used to define binary classes [19]. Performance metrics included the plotted ROC(t), the associated AUC(t) [20-22], and the p-value of a randomization test of its significance.

### Randomization test

The randomization test is a non-parametric test by permuting the survival times of the training data to generate the null dataset that patients’ survival times are not associated with the clinical and gene expression variables. The prediction model was fit to the null dataset, and performance metrics were computed on the test dataset and compared to the corresponding metrics calculated from the observed data. The procedure was repeated 10,000 times. The proportion of the estimated metrics calculated from the null dataset that exceeded the metric calculated from the observed dataset was the p-value of the randomization test. The metrics obtained by the randomization test were D_xy_, p-value of Cox model, R^2^ and AUC(t).

### Power validation

Cross-validation and bootstrapping are two methods commonly used to assess performance of a prediction model. Both methods are based on resampling techniques Cross validation involves repeatedly splitting the data into a training set and test set, where the training set is used for model development and the test set is for model validation and performance assessment. The predictive performance is the average of the numerous training-test partitions. In particular, a split sample validation refers to splitting the entire data into a training set and a test set, and only the test set is used to evaluate once without "crossing". Bootstrapping analyzes subsamples repeatedly, where each subsample is a random sample with replacement from the entire data. Various bootstrap methods such as the ordinary bootstrap, the leave-one-out bootstrap and the .632+ bootstrap are proposed and compared by Efron [39] and Efron and Tibshirani [40,41]. The power of the prediction models were evaluated by 2-fold cross validation [42], and the procedure was repeated 5,000 times. The proportion of p-values less than or equal to 0.05 were calculated as an estimate of the power.

### Assessment of binary classification of patients in test dataset

Binary classifiers for the five models with the same signatures selected from the risk prediction models were developed using the support vector machine (SVM), random forest classification (RF) algorithms, and logistic regression. These three algorithms are the most frequently used algorithms and have been shown to perform well in the analysis of microarray data. Performance metrics were the numbers of misclassified samples for each metastasis-free survival threshold.

## Results

The dataset of van 't Veer et al. [26] contained 78 primary breast cancers (34 from patients who developed distant metastases within 5 years (poor prognosis) and 44 from patients who continue to be disease-free (good prognosis) after a period of at least 5 years). The available clinical variables included age, diameter, tumor grade, angioinvasion, oestrogen and progesterone receptor status, and lymphocytic infiltration. The 78 patients were used as training data to develop prediction models; an additional 19 patients including 7 with good prognoses and 12 with poor prognoses were used as test data. Although this dataset is small, its size represents many existing datasets that have a cancer-related endpoint as the outcome variable with many genes as predictor variables.

### Results of survival prediction

Table 1 shows the estimates of performance metrics for the five models from two fittings of the Cox model. Model I used the risk scores as an independent variable (Columns 3–7). Model II used the risk groups (high versus low risk group) as an independent variable (Columns 8–12), based on the median of the training scores. Table 1 also shows the calculated values of Somers’ rank correlation coefficient, D_xy_. The predicted risk scores and risk rankings are shown in Additional file 1: Table S2.

**Table 1:** **Performance metrics of the five prediction models for the breast cancer data: Somers’ correlation (D**_**xy**_**); estimates of the hazard ratio (HR) with 95% confidence limits (CI), and p-value for Cox Models I and II; and R**^**2**^**for Cox Model I and Brier score (IBS) for Cox Model II**

**Model**	**D**_xy_	**Cox Model I**					**Cox Model II**				
		**R**^2^	**HR**	**95% C.I.**		**P**	**HR**	**95% C.I.**		**P**	**IBS**
A	−0.333	0.311	2.37	1.21	4.62	0.012	4.85	1.38	17.04	0.014	0.124
B	−0.310	0.114	1.71	0.87	3.39	0.123	1.99	0.75	5.27	0.167	0.148
C	−0.099	0.009	1.08	0.75	1.56	0.669	2.83	1.01	7.94	0.048	0.136
D	−0.310	0.248	2.50	1.12	5.57	0.026	2.36	0.82	6.76	0.111	0.144
E	−0.111	0.058	1.24	0.84	1.83	0.280	2.83	1.01	7.94	0.048	0.136

**Figure 1:**
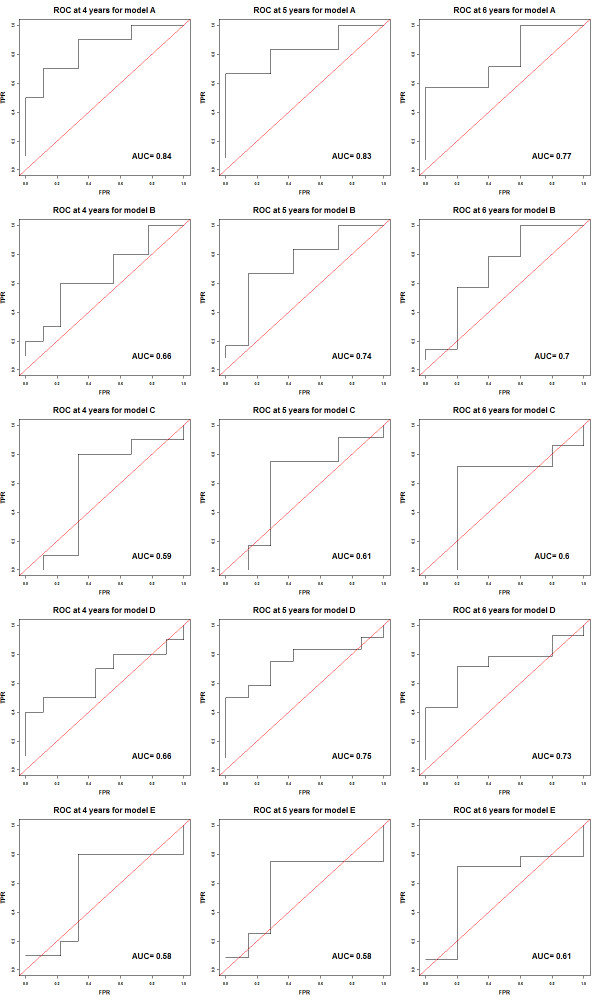
ROC curves for patients’ survival with AUC measures evaluated at 4, 5, and 6 years metastasis-free times for the five models.

Additional file 1: Figure S1 shows the Kaplan–Meier survival curves with the p-values from the log-rank test, and the Brier scores of the five models for each follow-up time points are also shown in Additional file 1: Figure S2. As expected, the p-values from the log-rank test and Model II analysis are very close. ROC and AUC analyses at the 4, 5, 6 metastasis-free times for the five models show similar results to one another (Figure 1).

The performance estimates obtained from Models I and II appear to be inconsistent for Models C and D (Table 1). Model C shows a small HR estimate and small absolute value of D_xy_ from the Model I analysis, but a significant HR estimate from the Model II analysis, while Model D shows the opposite. In all analyses, Model A has the smallest p-values and the largest absolute D_xy_, R^2^, and AUC for all three time points. The estimates for D_xy_, R,^2^ and AUC are useful for comparison between two prediction models, but the actual values, such as D_xy_ of −0.333, R^2^ of 0.311, and AUC of 0.84, are uninformative to infer the significance of the prediction.

The randomization (permutation) test was used to assess statistical significance of the observed performance measures shown in Table 1 and Figure 1, including the significance of the p-value of the hazard ratio itself. The randomization test generated the null distribution of no association (no predictability) between the 78 training patients and 19 test patients to assess the significance of the risk scores predicted by a prediction model. We illustrated an analysis for D_xy_, P-value, R^2^ and AUC metrics for Model I. In the permutation test, the survival times of the 78 patients were randomly permutated to generate a null dataset. Five prediction models, A-E, were developed from the null dataset. In each of the five models, two predictive models were developed. M1 was developed using the same signature predictors developed from the original 78 patient training dataset. M2 was developed by generating new predictors based on the null dataset. In M1 the null distributions were generated conditionally on the same signature, while in M2 the null distributions were generated unconditionally. The null hypothesis under both models was that the signature developed does not associate with the test data. Each prediction model was applied to the 19 test patients; the performance metrics D_xy_, P-value, R^2^ and AUC evaluated at 4, 5, and 6 year metastasis-free times were estimated. The procedure was repeated 10,000 times to generate the null distributions of the metrics. The proportion of the estimated metrics calculated from the null dataset that exceeded the metric calculated from the observed dataset was the p-value of the randomization test shown in Tables 2 and 3. In Model A, the p-values from M1 and M2 are identical in Table 2 since both models used all clinical variables.

**Table 2:** **P-values of randomization test based on 10,000 permutations for the three measures: Somers’ correlation (D**_**xy**_**), p-value of the hazard ratio, and R**^**2**^**from fitting the Cox proportional hazards model using the risk scores as independent variable**

**Model**	**M1 (Original predictors)**			**M2 (Re-select predictors)**		
	**D**_xy_	**P**	**R**^2^	**D**_xy_	**P**	**R**^2^
A	0.0369	0.0225	0.0261	0.0369	0.0225	0.0261
B	0.0707	0.3622	0.3802	0.0499	0.1747	0.1788
C	0.2684	0.6027	0.6084	0.3146	0.6761	0.6838
D	0.0487	0.0718	0.0661	0.0387	0.0445	0.0383
E	0.2534	0.3170	0.3255	0.2588	0.3078	0.3166

**Table 3:** P-values of randomization test based on 10,000 permutations for the AUC measures evaluated at 4, 5, and 6 years metastasis-free times

**Model**	**M1 (Original predictors)**			**M2 (Re-select predictors)**		
	**4-year**	**5-year**	**6-year**	**4-year**	**5-year**	**6-year**
A	0.1134	0.0206	0.0508	0.1123	0.0195	0.0508
B	0.2692	0.2762	0.1293	0.2369	0.1898	0.0785
C	0.3786	0.2523	0.1920	0.4024	0.3083	0.2680
D	0.2243	0.2109	0.1020	0.1687	0.1503	0.0635
E	0.3686	0.3443	0.2991	0.3470	0.3007	0.3227

In Table 2, the p-values estimated from the randomization test for the metrics P-value and R^2^ are very similar since both metrics measure the association under the Cox model. For Model B, the randomization test p-values for D_xy_ and R^2^ appear to be different. The results from the left panel (original predictors) and right panels (re-selected predictors) are similar. In general, the p-values from the randomization test generally agree with the results based on asymptotic parametric tests from Model I of Table 1. The p-values for the AUC metrics in Table 3 are generally higher than the p-values for the metrics in Table 2. The AUC test was significance only at the 5-year metastasis-free time (for Model A). In both M1 and M2, Model A has the smallest p-values for D_xy_, P, R^2^ and AUC at all three time points.

A two-fold cross validation was used to estimate the power to detect an association between a prediction model and survival time. First the 78 and 19 patients were pooled. The 97 total patients were randomly split into a training set of 49 patients and a test set of 48 patients. Five prediction models were developed from the training set and then applied to the test set. (Since the original signature of each model was developed based on the 78 test samples, the original signature was no longer applicable in this analysis.) In cross-validation, the p-values were computed using the Cox models I and II, and the log-rank test. The procedure was repeated 5,000 times. The proportion of p-values less than or equal to 0.05 were calculated as an estimate of the power. The results are shown in Table 4. The results agree qualitatively with the results from the randomization test. Again, Model A appears to perform the best. Box plots of the empirical distributions of the p-values are given in Additional file 1: Figure S3.

**Table 4:** The 97 total patients were randomly split into a training set and a test set

**Model**	**Single Group Analysis**	**High- versus Low-Risk Group Analysis**	
	**Cox Model I**	**Cox Model II**	**Log-rank test**
A	0.7630	0.5032	0.5164
B	0.5408	0.4628	0.4778
C	0.2876	0.2844	0.2964
D	0.5810	0.4286	0.4382
E	0.3302	0.2600	0.2702

The numbers of patients for the training and test sets were 49 and 48, respectively. The values are the proportion that the estimated p-values were less than or equal to 0.05 from a total of 10,000 computations, based on 5,000 randomly splits.

We further investigated the effect of the training and test set sizes on the power estimation. The numbers of patients for the training set investigated were 78, 65, 32, 25, and 19. Only the results from Model A are shown (Table 5). The results from other models are given in Additional file 1: Table S3-S7. Table 5 shows that (78:19) and (19:78) are among the poorest performance. It appears that a small test sample size (78:19) will reduce the power. Steyerberg [43] also discusses this issue. On the other hand, a small training size (19:78) may affect the fitting of the model. The 2-fold or 3-fold cross validation can be used for power performance evaluation.

**Table 5:** Effect of training and test set sizes on the power for Model A

**Training: Test**	**Single Group Analysis**	**High- versus Low-Risk Group Analysis**	
	**Cox Model I**	**Cox Model II**	**Log-rank test**
78:19	0.3945	0.2623	0.2819
65:32	0.6000	0.4164	0.4312
49:48*	0.7630	0.5032	0.5164
32:65	0.7746	0.5226	0.5294
25:72	0.7042	0.5166	0.5232
19:78	0.563	0.4058	0.412

* From Table 4.

The 97 total patients were randomly split into a training set and a test set. The numbers of patients for the training set investigated were 78, 65, 32, 25, and 19. The values are the proportion that the estimated p-values were less than or equal to 0.05 from a total of 10,000 computations, based on 10,000 randomly splits.

### Binary classification and survival-time prediction

#### Binary classification

The breast cancer dataset was first presented to develop a binary classifier of 70 signature genes based on 5-year distant metastases [26]. The classifier misclassified 2 of the 19 test patients using both optimal accuracy and sensitivity threshold strategies. For the 19 patients in the test dataset, there were four patients (11, 12, 13, and 14) who had metastasis-free times between 4.77 and 5.23, around 5 years. We illustrate the analysis using 4-year, 5-year, and 6-year metastasis-free times to define high and low risk groups.

Table 6 shows the numbers of misclassifications using the thresholds of 4-year, 5-year, and 6-year metastasis-free times. The classification errors can be very different if different thresholds are used. Among the five models, Model B, as binary classifier, appears to have the best overall performance. The logistic regression performs better than the SVM and RF in model A. For the SVM algorithm, the numbers of misclassification errors based on the 4-year, 5-year, and 6-year survival thresholds are 8, 3, and 3, respectively; the numbers are 7, 3, and 2 for the RF algorithm; the numbers are 7, 7, and 4 for logistic regression. The misclassified patients by the three algorithms are given in Additional file 1: Table S8. The misclassification errors between 4-year and 5-year differ substantially in SVM and random forest.

**Table 6:** The numbers of misclassifications for five binary classifiers using the support vector machine (SVM), random forest (RF) and logistic regression (LR) classification algorithms

	**Survival Threshold**	**Risk**	**Number of Training**	**Number of Test samples**	**Number of Misclassification**				
					**A**	**B**	**C**	**D**	**E**
	**Year**	**Group**	**samples**						
SVM	4	high	28	10	10	6	7	7	7
		low	50	9	1	2	3	2	3
	5	high	34	12	9	1	3	2	5
		low	44	7	1	2	2	2	2
	6	high	43	14	5	2	3	2	2
		low	35	5	1	1	2	1	1
RF	4	high	28	10	10	6	5	5	7
		low	50	9	2	1	3	1	3
	5	high	34	12	10	1	5	5	6
		low	44	7	1	2	2	0	2
	6	high	43	14	6	1	5	3	6
		low	35	5	2	1	2	1	2
LR	4	high	28	10	7	5	6	6	6
		low	50	9	1	2	3	4	4
	5	high	34	12	8	3	6	7	8
		low	44	7	0	4	2	3	1
	6	high	43	14	6	1	3	3	7
		low	35	5	0	3	2	2	1

The binary classifiers are developed based on the 4-year, 5-year, and 6-year metastasis-free times to define the high and low risk classes.

### Survival-time prediction

**Figure 2:**
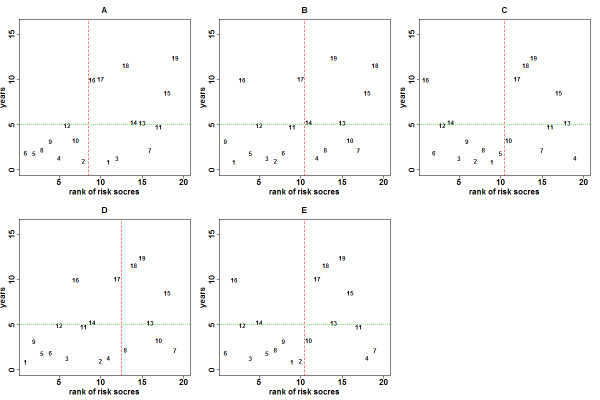
**Plot of metastasis-free survival time (vertical axis) of the 19 test patients versus the rank of the estimated risk score (horizontal axis) from the five risk prediction models.** The patients were numbered according to the ranks of their survival times. The patients on the left have high estimated risk scores (low ranks) and on the right have low estimated risk scores. Performance of a risk prediction model can be assessed by analyzing the relationship between survival times and risk scores (see text). For example, the horizontal line represents a cutoff at the 5 year metastasis-free time and the vertical line is the median of the training scores. A ROC curve can be constructed by enumerating all 19 vertical cutoffs and AUC can be computed (Figure 1).

**Figure 3:**
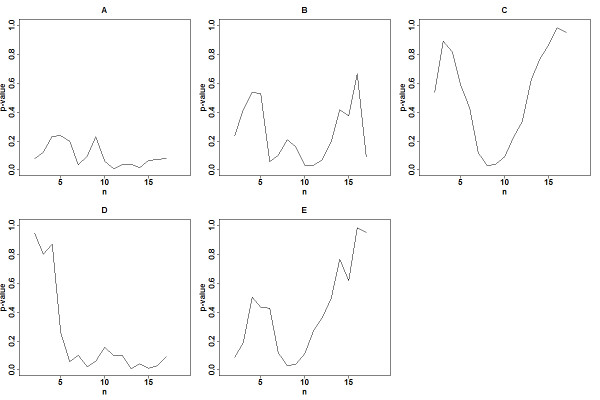
Loge-rank p-values of the five models for all possible sizes of low-risk group.

Although a survival prediction model is developed to predict survival risks of patients based on their predictor profiles, it can also be used as a binary prediction model. Figure 2 shows the plot of patients’ survival times and their ranked predicted risks for the five survival prediction models, where patients are ranked according to their survival times (Additional file 1: Table S2). Thus, Patient #1 (at the top) had the shortest survival time and Patient #19 (at the bottom) had the longest survival time. The horizontal axis represents the patient’s rank according to the estimated risk score from a prediction model, where a rank of 1 corresponds to the highest estimated risk score, etc. The patients on the left have high risk scores and on the right have low risk scores. For example, in Model A, Patient #6 (ranked 1st) has the highest estimated risk score and Patient #19 (ranked 19th) has the lowest estimated risk score. The vertical line is the median of the training scores that separate the patients into the high and low risk groups for a two-group comparison. This separation into two groups implies a binary classification. In fact, the ROC approach relies on an induced binary classification at each risk-score cutoff. With a ROC approach at the 5-year metastasis-free time (Figure 2, horizontal line), the patients on the upper left region have longer survival times but are categorized in the high risk group; the patients on the lower right region have shorter survival times but are categorized in the low risk group. Thus, Patients #1, #3, #7, and #11 are misclassified in Model A. Different horizontal lines can be plotted to evaluate predictive performance for different time points. The ROC curves constructed by enumerating all 19 vertical cutoffs with the AUC measure are shown in Figure 1. The ROC is a line connecting all the points without smoothing, and there are fewer jumps than 19 because some of the 19 points have same true positive rate or false positive rate. Figure 3 shows plots of p-values of the log-rank test using all possible cutoffs for Models A-E. The minimum p-values occur generally in the range when the numbers of patients in the low-risk group are between 8 and 11. It also indicates that the dichotomization of the survival risk scores into two groups to evaluate predictability could lead to different conclusions if different thresholds are applied.

In summary, a binary classifier is developed from a training dataset where each patient is pre-assigned into either a low-risk or high-risk group, while a survival risk prediction model is developed, based on the patients’ survival times without pre-assigning the patients into two groups. A binary classifier predicts a new patient as either high or low risk. A risk prediction model provides an estimate of risk score of a new patient; the estimated risk score can be compared with the median of the training scores to determine the patient’s risk group (high or low). The main deficiency in the use of a binary classifier to analyze survival time is the presence of censored observations. However, if there is no censoring, and the purpose is to classify patients’ survival risks at a specific time point of interest, then a binary classifier should be more powerful than a survival prediction model.

## Discussion and Conclusions

The development of prediction models using the cancer TNM staging system combined with the basic clinical covariates and microarray gene expression variables for identifying high-risk and low-risk patients for treatment recommendations has been an important goal in clinical oncology research. Several recent publications have investigated the use of microarray gene expression data to improve accuracy in estimating patient risk. However, the use of prediction models for clinical decision making still has many challenges to be overcome. A recent critical evaluation of published studies on lung cancer found little evidence that any of the reported gene expression signatures are ready for clinical application [21].

A prediction model is developed to predict survival risk of new patients which may come from different medical centers or different times. The ability to predict patients from different centers involves many factors, such as study protocols, microarray platforms, sample processing, and data pre-processing, etc. This study considers prediction of new patients assuming they are from the same study protocol. We focus on the assessment of performance of survival prediction models using five established prediction models.

Performance of a prediction model depends on the set of predictive signatures used in the model. Since the number of clinical variables is typically small, all clinical variables can be considered to develop a prediction model. On the other hand, since gene expression levels are often correlated, the set of predictors selected may vary substantially among different training samples, although the models predict about equally well [28]. It may not be feasible to come up with a general procedure to determine an optimal set of predictors (genes and clinical variables) for a “best” performance under the Cox model.

A common practice to assess performance of a survival risk prediction model is to evaluate its ability to separate the predicted risk scores of patients into low and high risk groups based on a particular cutoff threshold. However, the threshold has been defined differently; some researchers used the median or other percentiles of training scores as the cutoff [21,32-34] and others used the median or other percentiles of the test scores [35-37,44]. Different cutoffs to segregate the testing data could lead to different conclusions, and it also occurred in the binary classifiers such as SVM and random forest algorithm. A more fundamental issue is that a prediction model is developed, based on training of the available dataset, to predict new sample(s), classifying new patients as high or low risk based on the available data. Therefore, the median or other percentiles of the *training* scores should be used as a cutoff. In multiple center studies where a prediction model is developed from one center to predict patients of another center, comparing the medians of training and test scores will be useful to understand the underlying survival distributions of the two centers.

The survival time endpoint for risk prediction has been analyzed as a class prediction problem by dividing patients into two classes according to a survival-time threshold such as the breast cancer data [26]. The binary response approach provides natural performance metrics such as positive and negative predictive values to enable unambiguous assessments. The binary response approach addresses the question of whether the patient will survive up to a specific time, say, t*, while the survival-time risk prediction approach estimates the patient’s risk score. These two approaches address two different questions. The survival-time prediction approach is generally more appropriate and natural for modeling survival data in the presence of censored observations. This paper illustrates that binary classifiers highly depended on how the risk groups were defined. Binder et al. [30] investigated the effects of the choice of threshold on the predictions and showed that there is little overlap of selected genes between an early and median threshold cutoffs, which might be due to short-term and long-term effects of genes or the censoring pattern.

Performance of a risk prediction model is assessed by analyzing the relationship between survival times and risk scores. Many ROC studies mainly address a specific time point of interest [11,45,46]. Sun et al. [36] and van Belle et al. [47] showed time varying AUC measures for two different models to show an improvement of using gene expression data for predicting lung cancer survival, but the AUC measure from one model may not be consistently higher than the AUC measure from the other model across all time points. The assessment of the ROC curves for all time points might be needed. However, this can be impractical. Although accuracy comparison method developed by Moskowitz and Pepe [46] could be useful to assess performance among different models, this measure itself is inapplicable to assess the performance of a single model.

The Somers' index D_xy_ is a correlation measure for an overall concordance between predicted risk scores and observed survival times for the test data [11,44,48-52]. A high correlation implies that the predicted patients’ risk scores are in good concordance with the patients’ survival times. In most studies that presented D_xy_ values [11,44,48-50], they were used to show improvement of a new model [52] or to compare different models [34,53], without making inference to statistical significance. A few studies did report confidence limits [47,53]. Unlike R^2^, D_xy_ does not depend on the fitting of the Cox model.

Hielscher et al. [23] compared seven existing R^2^-type measures and showed their behavior in simulation examples and a gene expression microarray dataset. This paper evaluated several measures that have commonly been used for the evaluation in clinical oncology, including p-values of hazard ratios and logrank test, AUC, and three R^2^-type measures. A main conclusion in our analysis is that these existing metrics for evaluating the discriminatory ability of survival prediction models may lead to discordant results. In the lymphoma application, the seven R^2^-type measures reported in Table two of Hielscher et al. [23] were in agreement. They provided a summary of references of seven R^2^-type measures and available R software in Table three.

Cross validation of binary classifiers in gene expression data has been investigated extensively [54]. Cross validation of survival prediction models has not commonly been conducted. Recently, Subramanian and Simon [46] compared several re-sampling techniques for assessment of accuracy of risk prediction models, and their investigation covers various settings, including sample sizes, null model, number of k-fold partitions, etc. Although they only evaluated the AUC(t) at t = 180 months, they recommended 5- or 10-fold cross-validation which has good balance between bias and variability in the different settings. Simon et al. [14] also showed how to utilize cross-validation for the evaluation of prediction models using time dependent ROC curves. The cross validation to estimate power illustrated in this paper is similar to the approach used by Subramanian and Simon [46].

The p-values of the hazard ratios or log-rank test are commonly used to evaluate performance of risk prediction models. These p-values provide direct assessment of significance of the measures of predictability; however, some models can give inconsistent conclusions. D_xy_ measures an overall concordance between the patients’ survival times and predicted risk scores. AUC provides a probability measure of predictive ability at a given time point. The p-values of these two measures can be computed using the proposed randomization test, which cannot be derived theoretically. Both measures are very useful to assess performance of a single model or to compare different models.

## Competing interest

The authors declare that they have no competing interests.

## Authors’ contributions

JJC conceived the study and wrote the manuscript. HCC, RLK, and JJC improved the concepts. HCC developed and implemented the methodology and performed the analysis. RLK critically revised the context. KFC helped draft the manuscript. All authors read and approved the final manuscript.

## Authors’ information

The views presented in this paper are those of the authors and do not necessarily represent those of the U.S. Food and Drug Administration.

## Pre-publication history

The pre-publication history for this paper can be accessed here:

http://www.biomedcentral.com/1471-2288/12/102/prepub

## Supplementary Material

Additional file 1 Supplementary method. Click here for file
