# Supplementary material for: Assessment of performance of survival prediction models for cancer prognosis
Source: BMC Med Res Methodol. 2012 Jul 23;12:102. doi: 10.1186/1471-2288-12-102 (PMC3410808; doi:10.1186/1471-2288-12-102)

**Supplementary Method**

**The Hazard Ratio and Coefficient of Determination R^2^.** The estimated risk scores for the test data were fitted to a Cox model as an independent variable with survival time as the outcome variable. The exponent of the regression coefficient was the hazard ratio, which was the predicted rate of hazard in fitted model over the background that the estimated risk scores were uncorrelated with the survival times. The p-value and the confidence limits of the hazard ratio were measures of the predictive ability of the model.

The coefficient of determination R^2^, which represents the proportion of the variation explained by the fitted Cox model and is also known as pseudo R^2^, provides a measure of the goodness-of-fit of the predicted risk scores to the patient’s survival times [1]. It is equal to 1-exp(-LR/n) where LR=2log(L_U_/L_R_), and L_U_ and L_R_ are the restricted and unrestricted maximum likelihoods.

**P-values and Brier Scores for the High-Risk versus Low-Risk Group Comparison.** The test data were first segregated into two groups, high-risk and low-risk groups, by the median of the training scores; the univariate Cox model or log-rank test were applied to compare the difference in survival time between the two groups. The Cox model was fitted using the patient’s risk group (high-risk or low-risk group) as an independent variable. The hazard ratio here was the predicted rate of hazard between the two groups. The p-values of the Cox coefficient and log-rank test were evaluated for significance of the two group comparison. If the range of the scores between the training and test datasets differed substantially, then it might fail to separate the test data into two groups (all data in one group), or one of the two groups containing only censored data. In these cases, no comparison could be performed.

The Brier scores [2] for a specific time point in the absence of censored observations can be obtained by

, where I() is indicator function, and $T_{i}$ and $\hat{S}$ are survival time and estimated event-free probability for i-th patient having covariate $x_{i}$ at time t. If there are presences of censored observations, and the brier score can be given by

,

where $\hat{G}(t)$is the Kaplan-Meier estimate for the whole sample, and δ is binary variable to represent the presence (δ=0) or absence (δ=1) of censoring. Integrated Brier score (IBS) used to evaluate overall performance is defined as

$IBS=\frac{1}{t_{\max}}\int_{0}^{t_{\max}} BS(t)dt$, where $t_{\max}$is the maximum follow-up time.

**Somers’ Rank Correlation for Censored Data.** The concordance index (c-index) between predicted score and observed survival time in the testing data was computed by a rank correlation adjusted for censored time [3]. c-index represents the likelihood that one patient with higher prognostic score will survive longer than the one with smaller prognostic score. If this value is close to 0 or 1, it indicates that the risk scores are negatively or positively associated with observed survival times, respectively. Alternatively, c-index can be expressed equivalently as a correlation measure, known as the Somers’ D_xy_ rank correlation as D_xy_ = 2(c-index − 0.5) [4].

**Time Dependent receiver operating characteristic (ROC) curve and area under the ROC curve (AUC).** The ROC curve is usually used to interpret predictive ability for binary classifier. Heagerty et al. [5] developed two time-dependent ROC curves to characterize the diagnostic accuracy for censored survival outcomes. One estimated the true positive rate (sensitivity) and false positive rate (1-specificity) based on Kaplan-Meier estimator, but it might lead to negative probability inconsistency which might induce non-monotone ROC curve. The other one was developed from the weighted Kaplan-Meier estimator to avoid the problem [1]. We propose a ROC curve which would not have the problem of negative probability inconsistency because the numbers of true positive (TP), false positive (FP), false negative (FN), and true negative (TN) are estimated in advance. The censored patients in them were represented by the expected numbers estimated from Kaplan-Meier survival curve for the all patients but not conditional Kaplan-Meier survival used in the time-dependent ROC curve. For time t, $T_{j}$, $\delta_{j}$, and $H_{j}$ represented the survival time, censorship (1=event and 0= censor), and risk score for j-th patient, and the TP(t), FP(t), FN(t), and TN(t) at time t was given as

$$TP(t)=\sum_{j=1}^{n} I(T_{j}\leq t, \delta_{j}=1,H_{j}\geq c)+I(T_{j}<t, \delta_{j}=0,H_{j}\geq c)(1-\prod_{t_{j}<t_{k}\leq t} (1-\frac{d_{t_{k}}}{n_{t_{k}}}))$$

$$FP(t)=\sum_{j=1}^{n} I(T_{j}>t,H_{j}\geq c)+I(T_{j}=t,\delta_{j}=0,H_{j}\geq c)+I(T_{j}<t, \delta_{j}=0,H_{j}\geq c)\prod_{t_{j}<t_{k}\leq t} (1-\frac{d_{t_{k}}}{n_{t_{k}}})$$

$$FN(t)=\sum_{j=1}^{n} I(T_{j}\leq t, \delta_{j}=1,H_{j}<c)+I(T_{j}<t, \delta_{j}=0,H_{j}<c)(1-\prod_{t_{j}<t_{k}\leq t} (1-\frac{d_{t_{k}}}{n_{t_{k}}}))$$

$$FP(t)=\sum_{j=1}^{n} I(T_{j}>t,H_{j}<c)+I(T_{j}=t,\delta_{j}=0,H_{j}<c)+I(T_{j}<t, \delta_{j}=0,H_{j}<c)\prod_{t_{j}<t_{k}\leq t} (1-\frac{d_{t_{k}}}{n_{t_{k}}})$$

where $d_{t_{k}}$,$n_{t_{k}}$, c and $t_{k}$ were the number of events, the number of patients at risk at time $t_{k}$, the threshold of the risk scores, and the sorted unique follow-up time respectively. All of the four values were non-negative, and thus there was no negative probability estimator which could be present in the time-dependent ROC(t). Finally, the sensitivity and specificity for each unique follow-up time t can be estimated to construct ROC(t) curves. The AUC(t) is a summary measure of ROC(t), based on all risk score cutoff.

**Reference:**

1. Schemper M: **The relative importance of prognostic factors in studies of survival.** *Stat Med*, 1993, **12**:2377-2382.
2. Graf E, Schmoor C, Sauerbrei W, Schumacher M: **Assessment and comparison of prognostic classification schemes for survival data.** *Stat Med* 1999, **18**:2529-2545.
3. Harrell FE Jr, Califf RM, Pryor DB, Lee KL, Rosati RA: **Evaluating the Yield of Medical Tests.** *JAMA* 1982, **247**: 2543-2546.
4. Newson R: **Confidence intervals for rank statistics: Somers' D and extensions.** *The Stata Journal* 2006, **6**: 309-334.
5. Heagerty PJ, Lumley T, Pepe MS: **Time-dependent ROC curves for censored survival data and a diagnostic marker.** *Biometrics* 2000, **56**:337–344.

Table S1. The five risk prediction models

| Model | Predictors |
| --- | --- |
| A | clinical covariates model - Cox proportional hazards |
| B | gene expression model - 5 principal components |
| C | gene expression model - 10 most significant genes |
| D | clinical covariates-Cox + 5 principal components (A+C) |
| E | clinical covariates-Cox + 10 most significant genes (A+D) |

Table S2. Risk scores and rankings of the 19 test breast cancer patients estimated from the coefficients of fitted Cox proportional hazards model from training 78 training patients. The patient colored in red has the risk score corresponding to the median of the training scores.

| ID | Time | A | B | C | D | E |
| --- | --- | --- | --- | --- | --- | --- |
| 1 | 0.89 | -0.514(11) | 0.872(2) | 0.268(9) | 1.528(1) | 0.1(9) |
| 2 | 1.00 | 0.052(8) | 0.329(7) | 0.576(7) | -0.047(10) | 0.031(10) |
| 3 | 1.27 | -0.541(12) | 0.362(6) | 0.923(5) | 0.55(6) | 1.301(4) |
| 4 | 1.37 | 0.577(5) | -0.518(12) | -2.178(19) | -0.123(11) | -1.672(18) |
| 5 | 1.84 | 1.255(2) | 0.627(4) | 0.21(10) | 0.745(3) | 0.859(6) |
| 6 | 1.92 | 1.398(1) | -0.121(8) | 2.164(2) | 0.736(4) | 3.044(1) |
| 7 | 2.17 | -0.921(16) | -0.844(17) | -0.609(15) | -1.37(19) | -2.026(19) |
| 8 | 2.25 | 1.161(3) | -0.522(13) | 0.39(8) | -0.383(13) | 0.564(7) |
| 9 | 3.2 | 0.599(4) | 1.203(1) | 0.858(6) | 0.884(2) | 0.383(8) |
| 10 | 3.29 | 0.109(7) | -0.815(16) | -0.011(11) | -0.979(17) | -0.42(11) |
| 11 | 4.77 | -1.147(17) | -0.149(9) | -0.647(16) | 0.21(8) | -1.666(17) |
| 12 | 4.95 | 0.419(6) | 0.545(5) | 2.066(3) | 0.568(5) | 1.395(3) |
| 13 | 5.23 | -0.867(15) | -0.661(15) | -1.117(18) | -0.933(16) | -1.018(14) |
| 14 | 5.3 | -0.802(14) | -0.336(11) | 1.36(4) | 0.135(9) | 1.286(5) |
| 15 | 8.54 | -1.634(18) | -0.859(18) | -0.882(17) | -1.043(18) | -1.202(16) |
| 16 | 9.98 | -0.215(9) | 0.851(3) | 2.833(1) | 0.384(7) | 1.45(2) |
| 17 | 10.1 | -0.465(10) | -0.152(10) | -0.412(12) | -0.355(12) | -0.545(12) |
| 18 | 11.59 | -0.678(13) | -1.04(19) | -0.458(13) | -0.842(14) | -0.651(13) |
| 19 | 12.43 | -1.687(19) | -0.598(14) | -0.554(14) | -0.855(15) | -1.162(15) |

Table S3. The 97 total patients were randomly split into a training set and a test set. The numbers of patients for the training and test sets were 78 and 19, respectively. The values are the proportion that the estimated p-values were less than or equal to 0.05 from a total of 10,000 computations, based on 10,000 randomly splits.

|  | Single Group Analysis | High- versus Low-Risk Group  Analysis | |
| --- | --- | --- | --- |
| Model | Cox Model I | Cox Model II | Log-rank test |
| A | 0.3945 | 0.2623 | 0.2819 |
| B | 0.2398 | 0.2132 | 0.2371 |
| C | 0.1427 | 0.1533 | 0.1728 |
| D | 0.3274 | 0.2632 | 0.2851 |
| E | 0.1906 | 0.1651 | 0.1812 |

Table S4. The 97 total patients were randomly split into a training set and a test set. The numbers of patients for the training and test sets were 65 and 32, respectively. The values are the proportion that the estimated p-values were less than or equal to 0.05 from a total of 5000 computations, based on 5,000 randomly splits.

|  | Single Group Analysis | High- versus Low-Risk Group  Analysis | |
| --- | --- | --- | --- |
| Model | Cox Model I | Cox Model II | Log-rank test |
| A | 0.6000 | 0.4164 | 0.4312 |
| B | 0.3402 | 0.3316 | 0.3496 |
| C | 0.2194 | 0.2092 | 0.2246 |
| D | 0.4610 | 0.3400 | 0.3574 |
| E | 0.3074 | 0.2246 | 0.2398 |

Table S5. The 97 total patients were randomly split into a training set and a test set. The numbers of patients for the training and test sets were 32 and 65, respectively. The values are the proportion that the estimated p-values were less than or equal to 0.05 from a total of 5,000 computations, based on 5,000 randomly splits.

|  | Single Group Analysis | High- versus Low-Risk Group  Analysis | |
| --- | --- | --- | --- |
| Model | Cox Model I | Cox Model II | Log-rank test |
| A | 0.7746 | 0.5226 | 0.5294 |
| B | 0.5678 | 0.4976 | 0.5036 |
| C | 0.2882 | 0.2412 | 0.2476 |
| D | 0.5128 | 0.3946 | 0.4026 |
| E | 0.2522 | 0.1922 | 0.1972 |

Table S6. The 97 total patients were randomly split into a training set and a test set. The numbers of patients for the training and test sets were 25 and 72, respectively. The values are the proportion that the estimated p-values were less than or equal to 0.05 from a total of 5,000 computations, based on 5,000 randomly splits.

|  | Single Group Analysis | High- versus Low-Risk Group  Analysis | |
| --- | --- | --- | --- |
| Model | Cox Model I | Cox Model II | Log-rank test |
| A | 0.7042 | 0.5166 | 0.5232 |
| B | 0.4846 | 0.4388 | 0.4446 |
| C | 0.2398 | 0.2218 | 0.2256 |
| D | 0.4164 | 0.3276 | 0.3348 |
| E | 0.1582 | 0.1522 | 0.1558 |

Table S7. The 97 total patients were randomly split into a training set and a test set. The numbers of patients for the training and test sets were 19 and 78, respectively. The values are the proportion that the estimated p-values were less than or equal to 0.05 from a total of 5,000 computations, based on 5,000 randomly splits.

|  | Single Group Analysis | High- versus Low-Risk Group  Analysis | |
| --- | --- | --- | --- |
| Model | Cox Model I | Cox Model II | Log-rank test |
| A(4377) | 0.563 | 0.4058 | 0.412 |
| B(4373) | 0.3152 | 0.3010 | 0.3058 |
| C(3887) | 0.1598 | 0.1418 | 0.1458 |
| D(3347) | 0.2026 | 0.1056 | 0.1538 |
| E(3682) | 0.1172 | 0.108 | 0.1086 |

Table S8. The misclassified patients for five binary classifiers using the support vector machine (SVM), random forest (RF) and logistic regression (LR) classification algorithms. The binary classifiers are developed based on the 4-year, 5-year, and 6-year metastasis-free times to define the high and low risk classes.

|  | Survival Threshold  Year | Risk  Group | A | B | C | D | E |
| --- | --- | --- | --- | --- | --- | --- | --- |
| SVM | 4 | high | 1,2,3,4,5,6,7,8,9,10 | 2,3,4,5,7,10 | 1,2,4,5,7,9,10 | 2,3,5,6,7,8,10 | 1,2,4,5,7,9,10 |
|  |  | low | 12 | 12,16 | 12,14,16 | 12,16 | 12,14,16 |
|  | 5 | high | 1,2,3,4,5,6,8,9,11 | 10 | 4,7,11 | 5,10 | 2,4,5,7,10 |
|  |  | low | 16 | 14,16 | 14,16 | 14,16 | 14,16 |
|  | 6 | high | 1,3,11,13,14 | 10,13 | 4,11,13 | 11,13 | 4,13 |
|  |  | low | 16 | 16 | 16,18 | 16 | 16 |
| RF | 4 | high | 1,2,3,4,5,6,7,8,9,10 | 2,3,4,7,8,10 | 1,2,4,5,7 | 2,3,7,8,10 | 1,2,4,5,7,9,10 |
|  |  | low | 12,16 | 12 | 12,14,16 | 12 | 12,14,16 |
|  | 5 | high | 1,2,3,4,5,6,8,9,10,11 | 8 | 1,4,5,7,11 | 2,5,8,10,11 | 1,2,4,5,7,11 |
|  |  | low | 16 | 14,18 | 14,16 | NA | 14,16 |
|  | 6 | high | 1,3,9,11,13,14 | 13 | 4,5,7,11,13 | 10,13,14 | 1,4,5,7,11,13 |
|  |  | low | 16,17 | 16 | 16,18 | 16 | 16,18 |
| LR | 4 | high | 1,2,3,4,5,7,10 | 4,6,7,8,10 | 1,2,4,5,7,10 | 4,5,6,7,8,10 | 4,5,7,8,9,10 |
|  |  | low | 12 | 12,16 | 12,14,16 | 11,12,14,16 | 12,14,16,17 |
|  | 5 | high | 1,2,3,4,5,7,10,11 | 6,8,10 | 1,4,5,7,10,11 | 4,5,6,7,8,10,12 | 2,4,5,7,8,9,10,11 |
|  |  | low | NA | 14,16,17,19 | 14,16 | 14,16,17 | 14 |
|  | 6 | high | 1,3,7,11,13,14 | 10 | 4,11,13 | 7,10,13 | 2,4,7,9,10,11,13 |
|  |  | low | NA | 16,17,19 | 16,18 | 16,17 | 16 |

Figure S1. Kaplan–Meier survival curves of the patients for the 19 test data for the five prediction models with the p-value from the log-rank test. Each patient was classified into the high- or low-risk group based on the median of the training scores developed from the training 78 training data.


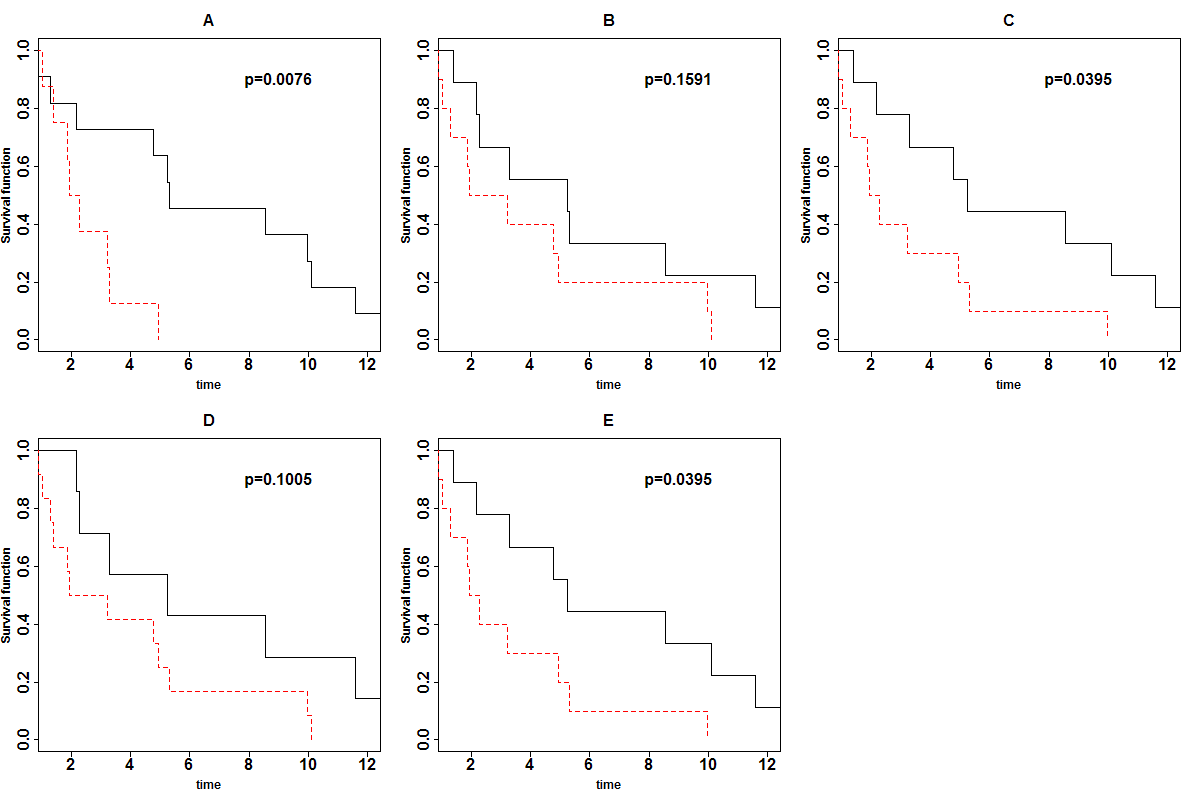


Figure S2. Brier scores of the five models for each follow-up time point in two-group comparison analysis


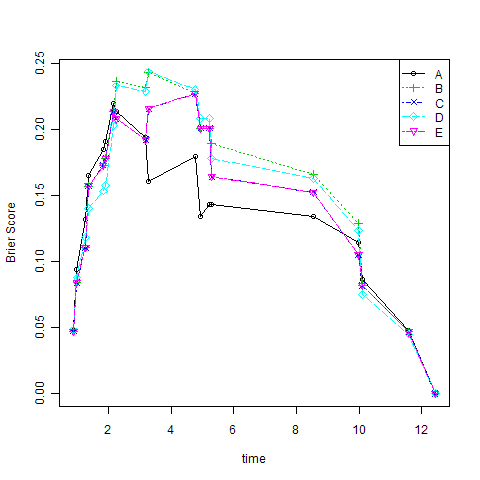


Figure S3. Box plots of the p-values from the cross validation using the Cox models I and II, and log-rank test. The p-values are –log_10_ transformed, the red line corresponds to the p-value at 0.05, -log0.05 = 1.3.
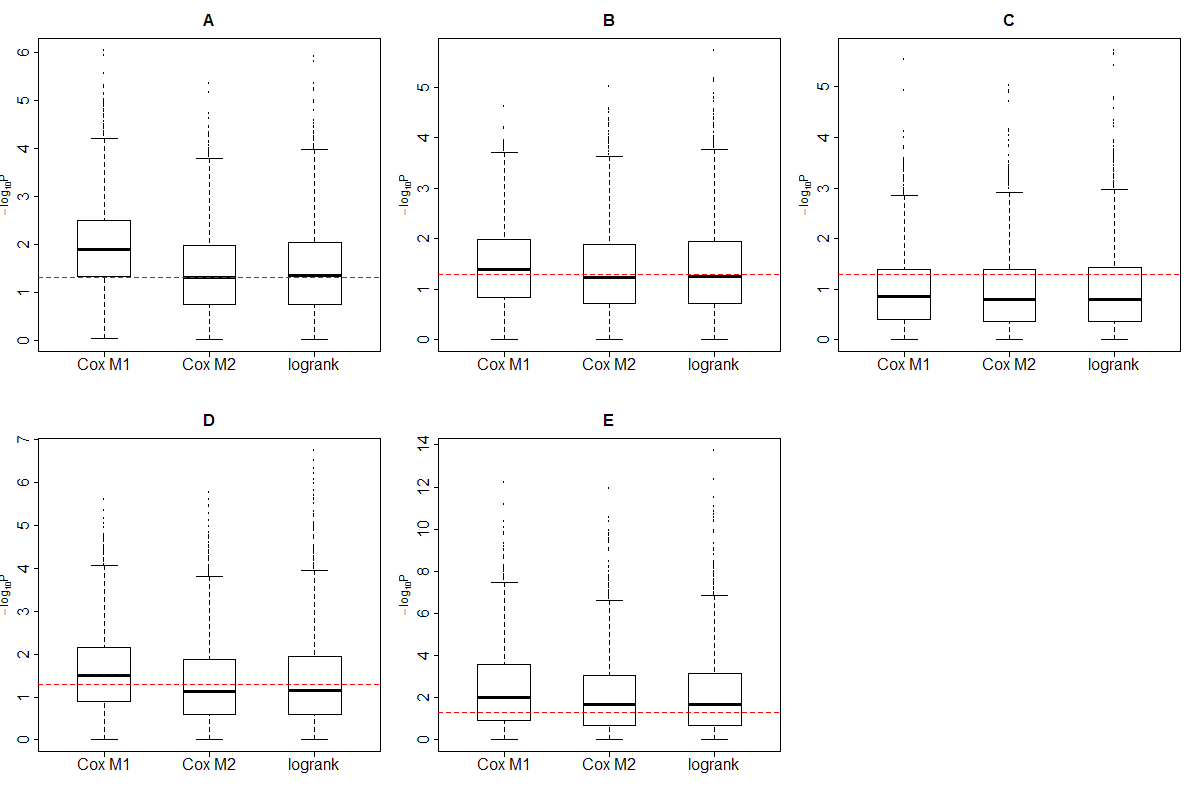

Supplement: Additional file 1 — Supplementary method. [file 1471-2288-12-102-S1.doc]
